# Treatment-related hemophagocytic lymphohistiocytosis due to atezolizumab: a case report and review of the literature

**DOI:** 10.1186/s13256-022-03585-3

**Published:** 2022-10-04

**Authors:** Jaime Rubio-Perez, Ángel Ricardo Rodríguez-Perez, María Díaz-Blázquez, Victor Moreno-García, Manuel Dómine-Gómez

**Affiliations:** 1 Medical Oncology Department, University Hospital Fundacion Jimenez Diaz, Instituto de investigación sanitaria FJD, Madrid, Spain; 2 Anatomic Pathology Unit Department, University Hospital Fundacion Jimenez Diaz, Madrid, Spain; 3 START Madrid-FJD, University Hospital Fundacion Jimenez Diaz, Madrid, Spain

**Keywords:** HLH, IrAE, Pharmacovigiliance, ICI

## Abstract

**Background:**

Immune checkpoint inhibitors avoid inhibition of T-cell responses, upregulating antitumor immune response. Moreover, a dysregulation with hyperactive immune response can be caused, some of them underdiagnosed. Hemophagocytic lymphohistiocytosis is a rare and often fatal syndrome of uncontrolled and ineffective hyperinflammatory response that triggers an inflammatory cascade that can lead in many cases to death.

**Case presentation:**

We report the case of a 67-year-old Caucasian man with stage IV lung adenocarcinoma who developed hemophagocytic lymphohistiocytosis after initiation of atezolizumab, an antagonist of programmed death-ligand 1. Even with early diagnosis and proper treatment, death occurs in approximately half of all cases reported.

**Conclusion:**

Key markers are needed to better identify patients at risk of developing severe immune-related adverse events. In addition to key markers, a higher degree of suspicion and early intervention are needed to improve outcomes in acquired hemophagocytic lymphohistiocytosis, especially with the increasingly and expanding use of immune activation.

## Background

It is now being seen, with the increasing number of tumor-related cases, that the use of immunotherapy is becoming more popular. An increase in the number of unknown immune-related adverse events (IrAEs) has been observed too, with secondary hemophagocytic lymphohistiocytosis (sHLH) being one of the most underrecognized and underreported of them all [1]. Immune checkpoint inhibitor (ICI) therapies exert their therapeutic effects by invigorating and stimulating an antitumor effect by CD8 T-cells. Off-target CD8 T-cell activation can trigger organ-limited damage or multiorgan failure as IrAE. Although the rate is low, in some cases this can be a life-threatening situation [2]. Besides, HLH syndrome is a hyperinflammatory reaction caused by uncontrolled activation of immune cells which results in a “cytokine storm” with progressive tissue injury and multiorgan dysfunction that, without treatment, causes death [3]. Depending on the etiology, it is classified as primary (due to genetic errors causing cytotoxic function defects) or secondary/acquired [1]. The different causes described in literature are immune dysregulation triggered by (mostly hematological) malignancies, different infections including novel severe acute respiratory syndrome coronavirus 2 (SARS-CoV-2), or autoimmune diseases. Furthermore, it is also called macrophage activation syndrome (MAS). Some cases are induced by drug reactions (including chemotherapy), while in others it is caused by a combination of the aforementioned factors [1, 3]. sHLH is well documented in a small number of case reports [4–25], and a cohort of individual safety reports in patients treated with immune checkpoint inhibitors [26].

Different clinical criteria have been described to identify HLH. In 1991, the International Histiocyte Society established the first criteria, which they prospectively validated from 1994 to 2004 but only for the pediatric population, thus these criteria were designed for primary HLH [27]. Since then, it has been assumed by consensus that these criteria can be applied to diagnose adult patients, too. When meeting five out of the eight criteria, the clinical diagnosis is highly probable. In 2014, a probability score based on a previous web-based, international Delphi study was described and retrospectively validated in adults, being named the H-score. This scale includes several clinical and analytical variables scored according to the value presented, giving a final score and associated probability of the syndrome [28]. Determining the H-score may be a preferable approach, and it can be easily obtained using an online calculator [29]. Potential cutoffs range between 138 and 169, the latter accurately classifying 90% of patients.

## Case presentation

We report the case of a 67-year-old Caucasian man who was reported to be an active smoker. His oncological history included immune thrombocytopenic purpura (ITP) as a paraneoplastic syndrome with 7000 platelets/mcL, associated with a 4.6-cm lung carcinoma as an incidental diagnosis. Diagnostic testing was performed, ruling out syphilis, hepatitis, and human immunodeficiency virus (HIV) infection, without gamma alteration in serum protein electrophoresis or any blood smear findings. No further autoantibody tests were performed. Corticosteroids were administered during 1 week at 1 mg/kg of methylprednisolone doses together with intravenous immunoglobulins during 5 days, with complete resolution of ITP.

After surgery, stage IIB (pT2aN1), moderately differentiated nonkeratinizing squamous cell carcinoma was confirmed. Surgical margins were affected, thus treatment was completed with concurrent weekly docetaxel with radiotherapy with good tolerance. Six months after initial presentation, early progression was evidenced with pleural thickening and pleural effusion. The pathological study was completed with no targeted mutations found and with programmed death-ligand 1 (PD-L1) <1% in tumor cells and 25% in stroma cells by 22C3 immunohistochemistry assay. First-line chemotherapy with carboplatin and paclitaxel was started, with partial response after receiving six cycles. Eighteen months after finishing first-line chemotherapy, with maintained response, new pleural progression was evidenced, as confirmed by positron emission tomography (PET) scan. It was thus decided to start a second line with atezolizumab (an anti-PD-L1 antibody).

Two weeks after receiving immunotherapy, he presented to the emergency room with severe dyspnea accompanied by intense asthenia, myalgia, and fever of 39 °C. He denied contact with people suffering coronavirus disease 2019 (COVID19) infection. Upon presentation, he was conscious and alert with all neurological functions preserved, hemodynamically stable, tachypneic, with 93% oxygen saturation in room air. He looked pale and had bruises on both arms on physical examination. Painless hepatomegaly and splenomegaly stood out, and auscultation revealed an abolition of left lung sounds. A blood test confirmed moderate pancytopenia, glomerular filtration rate of 50 ml/minute (previously normal), an increase in acute-phase reactants (C-reactive protein and ferritin), and elevated levels of D-dimer >20 times normal value (Table 1). A computerized angiotomography was performed with no signs of pulmonary thromboembolism, neither lung infiltrates nor pleural effusion.

During the next 24 hours, he started vomiting with an altered level of consciousness with drowsiness. He had miotic pupils, without other signs of neurological dysfunction. On examination, he had developed progressive jaundice with abdominal pain in the right flank. A few hours later, he suffered a tonic–clonic seizure.

**Fig. 1:**
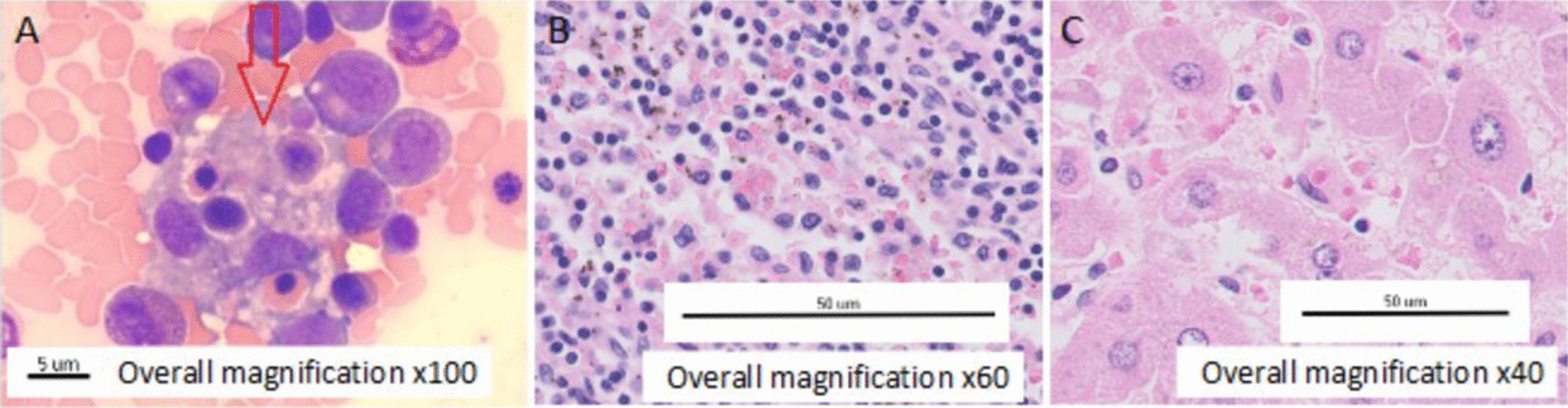
**A** Bone marrow biopsy with reactive hypercellularity, hemophagocytosis (red arrow), and dysplastic signs in megakaryocytic and erythroid reactive lines, confirming hemophagocytic syndrome. **B**, **C** Hepatic and spleen tissue with hemophagocytosis

Initially, COVID-19 was rejected, and blood tests were repeated at 24 hours, revealing pancytopenia, hyperbilirrubinemia, and hypertransaminitis. Head computed tomography (CT) was performed with no alterations, and a blood smear showed anisocytosis with some elliptocytes. The patient was admitted to the intensive care unit (ICU) and intubated due to neurological deterioration with a score of 8 on the Glasgow scale. After assessing the risks and benefits, no lumbar puncture was done. Empirical broad-spectrum antibiotic treatment was started, but the possibility that it was a hemophagocytic syndrome was raised and a differential diagnosis was started. Serologies were negative for HSV1-2, CMV, EBV, HIV, HBV, HCV, and HLTV-1 viruses. Elevated IL-6 (19.4 pg/mL) and CD25 (6516 U/m) were found with normal angiotensin-converting enzyme levels. He had 110 CD4 and 620 CD8 lymphocytes with very low CD4/CD8 ratio of 0.18. H-score for hemophagocytic syndrome was 256 points (> 99% probability). Given the high suspicion, treatment with high-dose corticosteroids was initiated using 20 mg dexamethasone bolus as 1 mg/kg equivalent of methylprednisolone since day 1. Finally, bone marrow biopsy was performed, showing reactive hypercellularity, hemophagocytosis, and dysplastic signs in megakaryocytic and erythroid reactive lines, thus confirming hemophagocytic syndrome (Fig. 1A).

Severe pancytopenia persisted despite transfusions, so it was decided to initiate tocilizumab at 8 mg/kg, without success, so we continued with anakinra without analytical or neurological improvement during day 2. Treatment with mycophenolate mofetil was added on day 3 for the next two days and finally, 5 days after admission, etoposide 100 mg/m^2^.

**Fig. 2:**
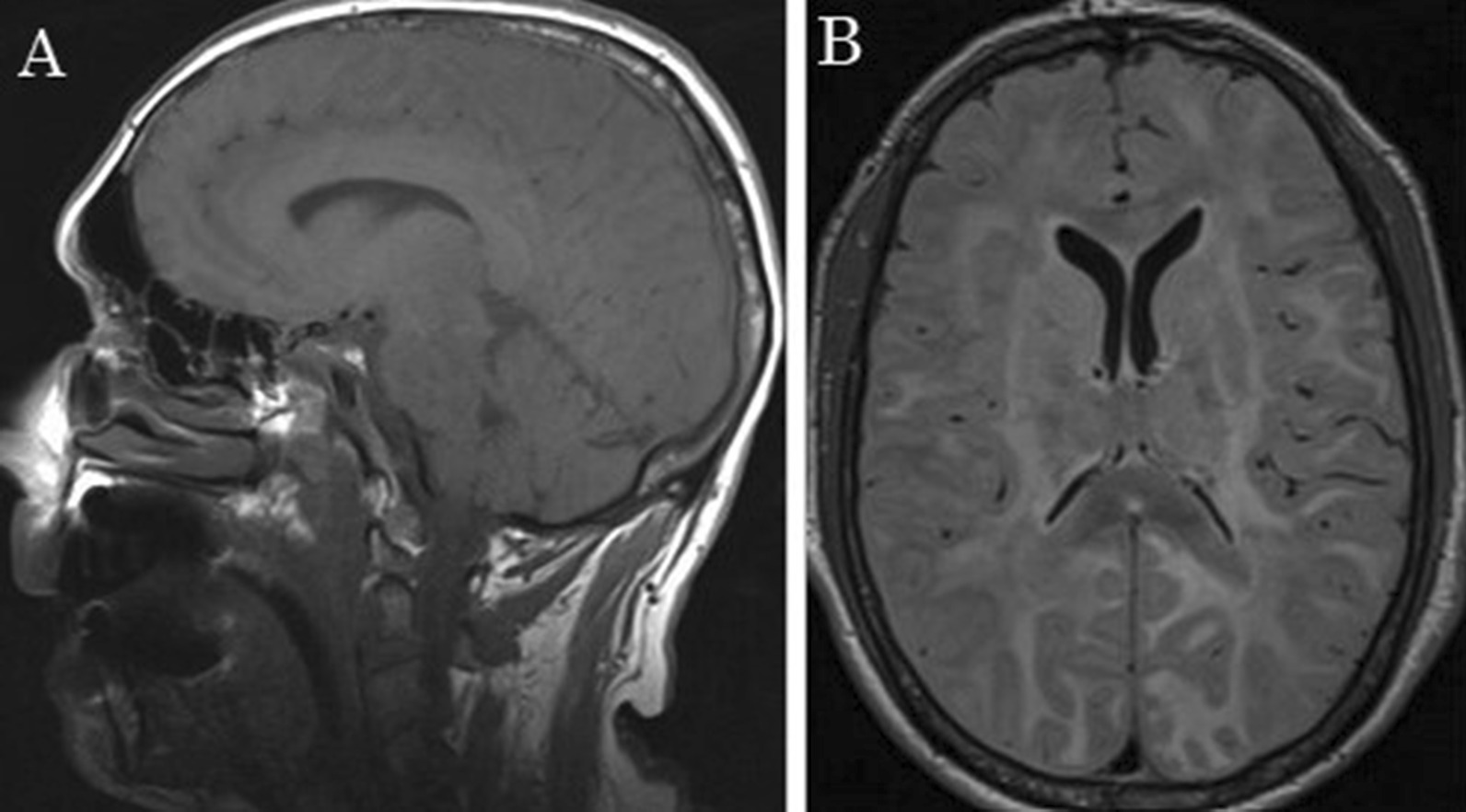
MRI T1 and T2 sequences with extensive involvement of supra- and infratentorial white matter, with bilateral and asymmetric damage, deep and superficial, of corpus callosum and internal capsules. **A** sagittal plane. **B** axial plane

Without any clinical improvement, magnetic resonance imaging (MRI) was performed, showing extensive white matter damage (Fig. 2). To study the degree of brain involvement, an electroencephalogram was done and revealed symmetrical background activity consisting of a 2–4 Hz, bilateral, irregular, low-voltage rhythm with no reactivity to eyelid closure–opening, corresponding to diffuse and severe degree brain involvement. Hence, it was decided to limit the therapeutic effort. Finally, the patient died 1 week after presentation.

Autopsy of the patient was performed, confirming the syndrome with hemophagocytic lymphohistiocytosis affecting the bone marrow, lymph nodes, liver, and spleen (Fig. 1B, C) as well as the central nervous system.

## Discussion

The major challenge in this case was the difficult differential diagnostic between HLH syndrome, an infection by different possible microorganisms including COVID-19 in the midst of the pandemic, together with ruling out other immune-related adverse events at the beginning of atezolizumab treatment. To the best of the authors’ knowledge, this is the first case report describing this rare systemic toxicity with sole use of atezolizumab as cancer therapy. Another case has been described, with the use of combination chemotherapy with carboplatin and nab-paclitaxel with atezolizumab, and the addition of autoimmune hemolytic anemia to sHLH [22]. As in the previous case report, our case is alarming due to the fast development of this deadly complication, after a single infusion of atezolizumab. One limitation is that we did not study initial autoantibodies for the study of the paraneoplastic ITPI presentation of our case, which could have shed light on the possible correlation between preexisting autoantibodies as biomarkers for the risk of developing hematological or other immune-related toxicities [30, 31].

Survival in adult malignancy sHLH ranges from 20% to 88%, considering refractoriness, secondary infections due to heavy immunosuppression, and progression of the underlying cancer disease [32]. Nevertheless, in the context of therapy-related sHLH with ICI, good outcomes have been reported only with the use of high doses of steroids [9, 11], probably at an early phase of sHLH and because of their use for other immune toxicities (Table 2) [33]. Major contributors to fatal outcome appear to be delayed administration of treatment because of poor recognition of unspecific symptoms and the presence of neurological involvement [3].

**Table 1:** Laboratory values along the clinical course, upon presentation, and after the different therapeutic strategies

	Presentation	Day 3 (48 hours after steroids)	Day 5 after MMF, anakinra, tocilizumab, and etoposide
White blood cells, k/cumm	2.58	3.08	1.92
Lymphocytes, k/cumm	1.2	2.3	0.7
Hemoglobin, g/dL	7.1	7.4	6.4
Reticulocytes (absolute)	0.0128		
Platelets, k/cumm	25	23	17
AST, U/mL	122		
ALT, U/mL	108	201	305
Creatinine, mg/dL	1.16	0.9	0.7
Total bilirubin, mg/dL	3.2	1.8	1.3
Indirect bilirubin, mg/dL	0.1	0.4	0.4
Triglycerides, mg/dL	151		
CRP, mg/dL	14.18	5.1	1.4
Ferritin, ng/dL	7035		
Fibrinogen, mg/dL	507	244	133
D-dimer	9042	8309	7357
sIL-2R (CD25), U/mL	6516		
IL-6, pg/dL	19.4		

*K/cumm* cells per microliter, *MMF* mycophenolate mofetil, *g/dL* grams per deciliter, *AST* aspartate aminotransferase, *U/mL* units per mililiter, *ALT* alaline transaminase, *mg/dL* miligrams per deciliter, *CRP* C reactive protein, *ng/mL* nanograms per mililiter, *sIL-2R* soluble interleukin-2 receptor, *CD25* cluster of differentiation protein 25, *IL-6* interleukin 6, *pg/ml* picograms per mililiter

**Table 2:** List of case reports of hemophagocytic lymphohistiocytosis in oncological patients with immune checkpoint inhibitors

Ref.	Immunotherapy	Primary tumor	Bone marrow biopsy	Neurology symptoms	Treatment	Clinical outcomes
[4]	Nivolumab	NSCLC	+	No	Steroids	Improvement
[5]	Nivolumab/ Ipilimumab/ Avelumab (3 cases, monotherapy)	Melanoma/ Merkel cell carcinoma	+/−	Unknown	Steroids	Death/ improvement
[6]	Pembrolizumab	Bladder carcinoma	+	Unknown	Steroids and etoposide	Unknown
[7]	Ipilimumab and nivolumab	Melanoma	Not done	No	Steroids and mycophenolate mofetil	Improvement
[8]	Pembrolizumab	Melanoma	Not done	No	Steroids	Improvement
[9]	Ipilimumab and nivolumab	Melanoma	+	No	Steroids	Improvement
[10]	BRAF inhibitors sequential after pembrolizumab	Melanoma	Not done	No	Steroids	Improvement
[11]	Ipilimumab sequential after pembrolizumab	Melanoma	+	No	Steroids and etoposide	Death
[12]	Pembrolizumab	NSCLC	+	No	Steroids	Improvement
[13]	Nivolumab	NSCLC	Not done	No	Steroids and mycophenolate mofetil	Improvement
[14]	Pembrolizumab	Thymic cancer	+	Yes	Steroids, IVIG, anakinra	Death
[15]	Pembrolizumab	Prostate cancer	+	No	Steroids+ plasmapheresis + etoposide + tacrolimus	Improvement
[16]	Pembrolizumab	Breast cancer	Not done	No	Steroids	Improvement
[17]	Nivolumab and anti-IDO	Glioblastoma	+	Yes	Steroids	Death
[18]	Pembrolizumab	Head and neck	+	No	Steroids and etoposide	Improvement
[19]	Pembrolizumab	NSCLC	+	No	Steroids	Improvement
[20]	Ipilimumab and nivolumab	Melanoma	+	No	Steroids, tocilizumab	Improvement[
[21]	Pembrolizumab	Pulmonary sarcomatoid carcinoma	Not done	No	Steroids	Death
[21]	Ipilimumab + nivolumab	Melanoma	Not done	No	Steroids + etoposide + IVIG + tocilizumab	Improvement
[21]	Ipilimumab + nivolumab	Melanoma	+	No	Steroids + etoposide	Death
[21]	Ipilimumab + nivolumab	Melanoma	−	No	Steroids	Improvement
[21]	Ipilimumab + nivolumab	Melanoma	+	No	Steroids	Improvement
[22]	Atezolizumab + chemotherapy	NSCLC	+	No	Steroids	Improvement
[23]	Pembrolizumab	NSCLC	+	No	Steroids	Improvement
[24]	Pembrolizumab	NSCLC	+	No	Steroids	Improvement
[24]	Pembrolizumab	NSCLC	Not done	No	Steroids	Improvement
[25]	Pembrolizumab	NSCLC	+	No	Steroids + etoposide	Improvement

*NSCLC* non-small cell lung cancer, *antiIDO* anti Indoleamine 2,3-Dioxygenase, *IVIG* intravenous immunoglobulins

By the time the HLH-2004 criteria are met, the patient may be beyond the point of optimal intervention. Furthermore, the H-score may be less specific for oncological patients, given baseline cytopenia and/or transaminitis due to previous therapy regimens, chemo-immunotherapy combination or metastasis, as could happen with organomegaly, too. Therefore, the trend among clinicians is to initiate therapy before traditional diagnostic criteria are met. Besides, the gradual introduction of new drugs based on clinical presentation and cytokine profile has resulted in the best adult survival rate reported in literature [34]

Activated immune effector cells and the local and systemic effects of inflammatory cytokines such interferon-gamma (IFN-g), tumor necrosis factor (TNF)-a, and interleukins (IL) 1b, 6, 8, 10, and 18 are responsible for the HLH pathogenesis [1]. Therefore, new strategies are under clinical development such as new targeted therapies such as ruxolitinib, a JAK 1-2 multicytokine receptor inhibitors [35] or drugs that block IL-1 (especially in the context of MAS [1, 33, 36]) or IL-6 [36].

Immune-activating therapies for cancer may induce a systemic IrAE named “cytokine release syndrome” (CRS), caused by overactivation of T-cells upon recognition of its target. The average time to CRS is the first week after administration of immunotherapy. This syndrome is characterized by the presence of pyrexia, tachycardia, hypotension, tachypnea, myalgia, transient confusion, delirium, aphasia, and seizures, among other symptoms mimicking sHLH in our case, and it is secondary to high amounts of TNF-a and IL-6 being released [37]. This differences in the cytokine profile between CRS and sHLH may be owing to differences in the immune cell subtypes stimulated and the different cytokines produced. Accordingly, new therapies focused against T-cells are being evaluated in this context, such as alemtuzumab, an antibody directed to CD-52 [38], or CD25.

Finally, we followed the latest guidelines regarding sHLH management [1, 27], which suggests that patients with severe active disease or neurological involvement, despite steroids, cyclosporine, or mycophenolate mofetil, and/or anakinra, may benefit from a reduced dose of etoposide (50–100 mg/m^2^ once weekly), to remove activated T cells and suppress inflammatory cytokine production [39]. We tried this, and added tocilizumab also, although it does not cross the blood–brain barrier, given the ICI trigger and the elevation of IL-6 found, but without any success. This may be due to the late diagnosis and the rapid worsening with extensive neurological involvement.

## Conclusion

With the increasing use of novel agents such as checkpoint inhibitors, the toxicity profile of drugs has changed and uncommon syndromes are more frequent nowadays, being more common in patients with comorbidity or treated using drug combinations. Recently, sHLH has been described in patients receiving ICI therapy more frequently. It is considered a rare adverse event, but may be underdiagnosed. It can develop from the first few weeks to months after treatment initiation and can occur at any time, even after discontinuation. Finding hemophagocytosis is neither pathognomonic nor required for diagnosis, and it is often not detected at initial presentation. Early intervention is critical to prevent progression and improve the patient's condition, so premature steroid initiation generally carries a favorable prognosis. In cases where there is no improvement with steroids, aggressive supportive management is necessary with intensification of therapy, following multi-immune suppressive drug protocols and adding interleukin-targeted therapies, with previous poor results reported. While the potential impact of such procedures is recognized, an optimal regimen sequence still has to be found, and the development of specific protocols for these patients is necessary.

## Data Availability

Information was obtained from the patient´s medical history records, images were from authorized investigations. Published reports were accessed from medical journals, and safety signals gathered from public access pharmacovigilance databases: Eudra (https://www.adrreports.eu/en/search.html), Vigibase (http://www.vigiaccess.org).

## Abbreviations

ICI: Immune checkpoint inhibitors
HLH: Hemophagocytic lymphohistiocytosis
PD-L1: Programmed death-ligand 1
IrAE: Immune-related adverse events
sHLH: Secondary hemophagocytic lymphohistiocytosis
MAS: Macrophage activation syndrome
ITP: Immune thrombocytopenic purpura
IFN-g: Interferon-gamma
TNF-a: Tumor necrosis factor
IL: Interleukin
